# Navigating the transcriptional roadmap regulating plant secondary cell wall deposition

**DOI:** 10.3389/fpls.2013.00325

**Published:** 2013-08-29

**Authors:** Steven G. Hussey, Eshchar Mizrachi, Nicky M. Creux, Alexander A. Myburg

**Affiliations:** Department of Genetics, Forestry and Agricultural Biotechnology Institute, University of PretoriaPretoria, South Africa

**Keywords:** secondary cell wall, transcriptional network, transcription factor, *Arabidopsis*, wood formation

## Abstract

The current status of lignocellulosic biomass as an invaluable resource in industry, agriculture, and health has spurred increased interest in understanding the transcriptional regulation of secondary cell wall (SCW) biosynthesis. The last decade of research has revealed an extensive network of NAC, MYB and other families of transcription factors regulating *Arabidopsis* SCW biosynthesis, and numerous studies have explored SCW-related transcription factors in other dicots and monocots. Whilst the general structure of the *Arabidopsis* network has been a topic of several reviews, they have not comprehensively represented the detailed protein–DNA and protein–protein interactions described in the literature, and an understanding of network dynamics and functionality has not yet been achieved for SCW formation. Furthermore the methodologies employed in studies of SCW transcriptional regulation have not received much attention, especially in the case of non-model organisms. In this review, we have reconstructed the most exhaustive literature-based network representations to date of SCW transcriptional regulation in *Arabidopsis*. We include a manipulable Cytoscape representation of the *Arabidopsis* SCW transcriptional network to aid in future studies, along with a list of supporting literature for each documented interaction. Amongst other topics, we discuss the various components of the network, its evolutionary conservation in plants, putative modules and dynamic mechanisms that may influence network function, and the approaches that have been employed in network inference. Future research should aim to better understand network function and its response to dynamic perturbations, whilst the development and application of genome-wide approaches such as ChIP-seq and systems genetics are in progress for the study of SCW transcriptional regulation in non-model organisms.

## Introduction

The bulk of plant biomass is comprised of secondary cell walls (SCWs), consisting of a cross-linked matrix of cellulose, hemicellulose and lignin biopolymers. The latter form the basic scaffold of fibers and vessels found in angiosperm xylem. In addition to providing mechanical support, SCWs facilitate critical biological processes, such as water and nutrient transport, anther dehiscence, silique shattering, plant organ movement and response to pathogens (Caño-Delgado et al., 2003; Mitsuda et al., 2005; Fratzl et al., 2008; Mitsuda and Ohme-Takagi, 2008). Candidate genes involved in the biosynthesis of SCWs have been studied in both woody and herbaceous model species (e.g., Brown et al., 2005; Mellerowicz and Sundberg, 2008). These structural genes are under strict transcriptional control during xylogenesis (Hertzberg et al., 2001; Schrader et al., 2004), highlighting the central role of transcription factors in this regard (Du and Groover, 2010). Understanding the regulation of SCW deposition is important because of (1) the widespread use of lignocellulosic biomass in pulp, paper and cellulose-derived products, (2) the potential of second-generation biofuel feedstocks such as short-rotation hardwoods (e.g., *Populus*, *Eucalyptus*) (Rockwood et al., 2008; Carroll and Somerville, 2009; Hinchee et al., 2010), and (3) the role of cell wall material in nutrition and health (Fincher, 2009; Doblin et al., 2010; McCann and Rose, 2010). However, the challenges to studying transcriptional regulation in non-model organisms impede the improvement of lignocellulosic biomass for fiber, raw cellulose and biofuels.

Considerable progress has been made in understanding how TFs regulate SCW structural genes. To this end, various model organisms (*Arabidopsis*, *Oryza*, *Populus*) (e.g., Kubo et al., 2005; Grant et al., 2010; Zhong et al., 2011a) as well as *Zinnia* and *Arabidopsis* (trans)differentiation systems (Fukuda and Komamine, 1980; Oda et al., 2005) have been instrumental. In the last decade, studies in *Arabidopsis* in particular have revealed the existence of an extensive transcriptional network regulating SCW deposition in vessels, fibers, anther endothecium and structures (replum, endocarp, valve margin) within the silique (reviewed in Yamaguchi and Demura, 2010; Zhong et al., 2010a). Whilst a considerable diversity of TF families participate in SCW transcriptional regulation, the most prominent families of TFs involved in this network appear to be the NAC (*N*AM/*A*TAF/*C*UC) and R2R3-type MYB (*MY*ELO*B*LASTOSIS) family proteins, both characterized by conserved N-terminal DNA-binding domains and diverse C-termini that participate in transcriptional regulation (Ooka et al., 2003; Dubos et al., 2010).

General structures of SCW transcriptional networks have been illustrated in a number of reviews, based on knowledge of *Arabidopsis* (Umezawa, 2009; Zhong and Ye, 2009; Caño-Delgado et al., 2010; Yamaguchi and Demura, 2010; Zhang et al., 2010; Zhong et al., 2010a; Wang and Dixon, 2011; Zhao and Dixon, 2011; Pimrote et al., 2012; Schuetz et al., 2013), and monocots (Handakumbura and Hazen, 2012). A few primary research articles also depict schematic representations of the *Arabidopsis* SCW network, incorporating data from *Populus* and limited knowledge of *Eucalyptus* and *Pinus* SCW transcriptional networks (Zhong et al., 2008a, 2011b; McCarthy et al., 2009). However, aside from Umezawa (2009) who focused on the cinnamate/monolignol pathway, these representations have not fully captured individual protein–DNA and protein–protein interactions reported in the literature. In addition, the regulatory dynamics of SCW transcriptional regulation are poorly understood compared to network structure (i.e., connectivity). Furthermore, the methodologies used to generate evidence lines for SCW network reconstruction have not been extensively reviewed. Here we comprehensively integrate and illustrate the complexity of known protein–DNA and protein–protein interactions in the *Arabidopsis* SCW transcriptional network. We discuss the roles of putative regulatory modules in the network, highlighting known and hypothetical balancing mechanisms that may influence network behavior. Finally, we provide a critical review of the methodologies currently used to infer SCW transcriptional networks and recommend approaches for increasing reliability in inferring SCW transcriptional network structure.

## Vascular patterning and differentiation

**Figure 1:**
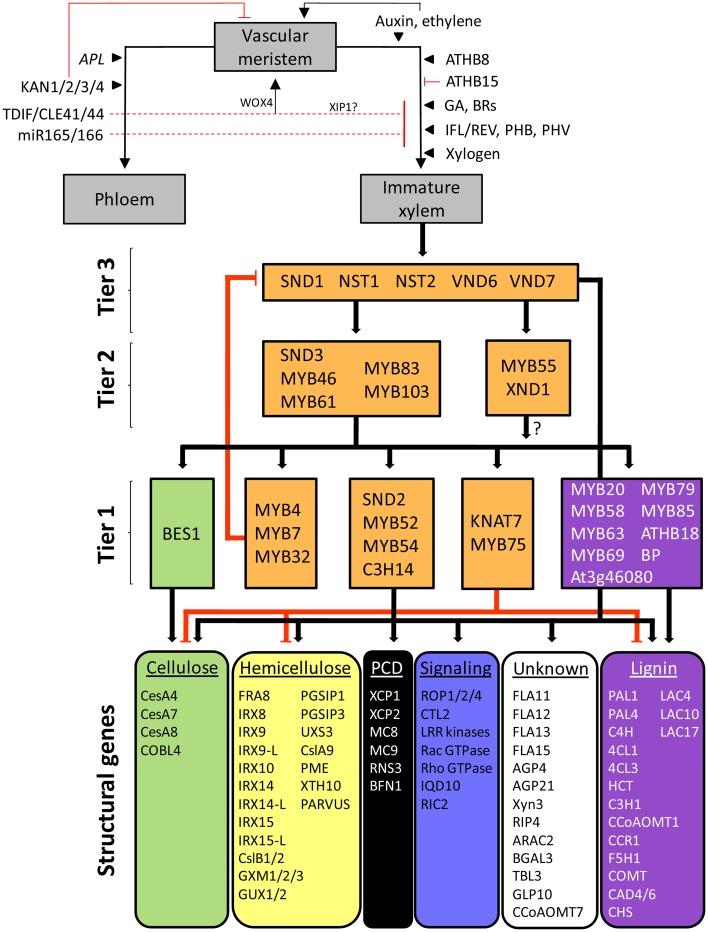
**The generalized *Arabidopsis* SCW transcriptional regulatory network in the light of vascular differentiation.** Vascular meristems, representing procambiums or secondary cambiums, produce mother cells that differentiate into phloem and immature xylem tissue (gray boxes) under the influence of transcriptional, hormonal, peptide, and miRNA regulators. Terminal differentiation of immature xylem cells into vessel elements and fibers is regulated by a tiered transcriptional network regulating genes associated with secondary cell wall cellulose, hemicellulose, programmed cell death (PCD), signaling, lignin, and genes with unknown functions. Positive regulation is indicated by black arrows; negative regulation is represented by red edges. Block colors represent different biological function categories. TFs currently known to regulate only one functional category are color-matched accordingly; orange blocks denote regulation of a combination of functional categories. The same color scheme is used in Additional file 1.

The deposition of SCWs and the initiation of programmed cell death (Bollhöner et al., 2012) together represent the culmination of developmental signals that cue vascular tissue specification and cell fate determination (Figure 1). This specification begins with establishing a population of meristematic cells known as the procambium via the combinatorial effect of hormones such as auxin, cytokinins and brassinosteroids (BRs). The procambium in turn gives rise to the primary vascular tissues (xylem, phloem) in the shoot vascular bundles and root vasculature (Turner et al., 2007; Caño-Delgado et al., 2010). In root and shoot tips, a pre-procambial state is established via PIN1-mediated polar auxin transport along files of parenchyma cells, effectively channeling auxin to what will become the procambium (Dettmer et al., 2009). In leaf veins, a preprocambial state is associated with expression of *ATHB8*, which is directly activated by the auxin response factor MP/ARF5 (reviewed in Zhang et al., 2010). In addition to procambium specification, auxin promotes cell division in the procambium in combination with cytokinins (reviewed in Caño-Delgado et al., 2010). The vascular cambium, from which all secondary xylem and phloem tissues arise during secondary growth, develops from the procambium and interfascicular parenchyma (Plomion et al., 2001; Baucher et al., 2007). As per the convention of Dettmer et al. (2009), we generally refer to procambiums and (secondary) vascular cambiums as vascular meristems, which are thought to be regulated in a similar, but not identical, fashion to shoot and root apical meristems (Sanchez et al., 2012; Milhinhos and Miguel, 2013) (Figure 1).

The establishment of xylem and phloem cell fate is influenced by hormones, TFs, miRNAs, mobile peptides and proteoglycans acting on nascent mother cells produced in the vascular meristems (Figure 1) (see Carlsbecker and Helariutta, 2005; Du and Groover, 2010; Zhang et al., 2010; Schuetz et al., 2013 for review). Auxin concentrations lower than those encountered at the vascular meristem promote xylem differentiation in the presence of cytokinin (Sorce et al., 2013). In the root, xylem differentiation is in contrast thought to be promoted by high auxin concentrations, brought about by cytokinin-mediated activation of a phosphorylation cascade in the procambium that results in polar auxin transport toward the protoxylem (reviewed in Aichinger et al., 2012). Five members of class III homeodomain leucine zipper (HD-ZIP III) TFs, including ATHB8, IFL1/REV, PHB, and PHV, are induced by auxin and generally promote xylem differentiation (Zhong et al., 1997; Baima et al., 2001; Ohashi-Ito and Fukuda, 2003; Ilegems et al., 2010; Schuetz et al., 2013). However, some HD-ZIP III genes, such as *ATHB8* and *ATHB15*, appear to be antagonistic to *REV* in meristem formation, embryo patterning and interfascicular fiber development (Prigge et al., 2005). For example, ATHB15 seems to negatively affect xylem development, while miR166-mediated cleavage of *ATHB15* transcript (see below) promotes xylem differentiation (Kim et al., 2005). Xylogen, a secreted proteoglycan, has also been implicated in xylem specification (Motose et al., 2004), while gibberellic acid (GA) promotes fiber elongation and general xylogenesis (Eriksson et al., 2000; Israelsson et al., 2003; Mauriat and Moritz, 2009). Brassinosteroids (BRs) have been associated with xylem differentiation in *Arabidopsis*, and in trans differentiating *Zinnia* cell cultures BRs are required for the expression of a homolog of *ATHB8* (reviewed in Jung and Park, 2007). Ethylene is essential for *in vitro* tracheary element (TE) differentiation in cultured *Zinnia* cells (Pesquet and Tuominen, 2011). *In planta*, ethylene is thought to diffuse from its site of synthesis in maturing TEs through to the cambium (Pesquet and Tuominen, 2011), where it promotes cell division (Love et al., 2009).

On the opposite side of the cambium, phloem differentiation occurs under the influence of APL, a MYB-related TF (Bonke et al., 2003; Ilegems et al., 2010), whilst KAN1/KAN2/KAN3/KAN4 TFs indirectly promote phloem differentiation by repressing (pro)cambium maintenance and restricting class III HD-ZIP TF expression through repression of polar auxin transport (Emery et al., 2003; Izhaki and Bowman, 2007; Schuetz et al., 2013). Phloem-expressed miR165/166, which are upregulated by SHR and SCR in roots, post-transcriptionally inhibit HD-ZIP III genes (Tang et al., 2003; Mallory et al., 2004; McHale and Koning, 2004; Zhong and Ye, 2004, 2007; Williams et al., 2005; Carlsbecker et al., 2010). Ectopic xylem formation is inhibited by a dodecapeptide ligand TDIF/CLE41/CLE44, which is produced in the phloem and diffuses to the xylem side of the vascular meristem (Ito et al., 2006). The peptide also co-ordinates the orientation of cell divisions in the cambium via the perception of a peptide concentration gradient by the LRR receptor-like kinase PXY in procambial cell membranes and induction of *WOX4* (Etchells and Turner, 2010; Hirakawa et al., 2010). Xylem differentiation may be further suppressed in the phloem in part by XIP1, which is related to PXY (Bryan et al., 2011) (Figure 1).

Once xylem mother cell fate has been established and cell elongation has ceased in immature xylem, SCW deposition occurs. This is activated by the TFs VND6 and VND7 in the case of xylem vessels, and SND1 and NST1 in fibers. These “master regulators” initiate a SCW transcriptional network, successively activating at least two tiers of intermediate TFs which, in addition to the master regulators, activate the structural genes for SCW biosynthesis (Figure 1, gray blocks). In the remainder of this review we focus on the SCW transcriptional network and the tools available to study its structure and function.

## The SCW transcriptional network: structure, evolution, and dynamics

**Figure 2:**
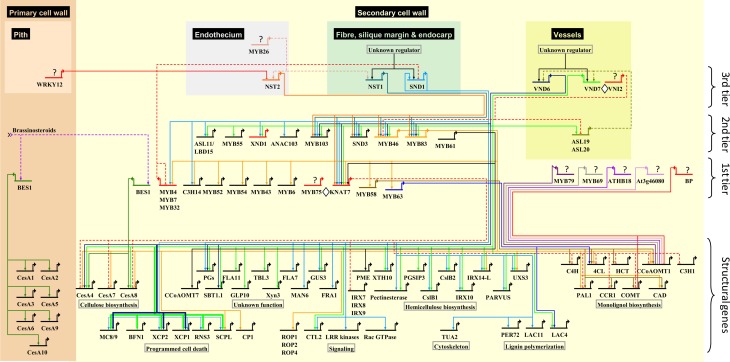
**Schematic representation of the protein–DNA interaction network underlying SCW biosynthesis in xylem fibers and vessels and anther endothecium in *Arabidopsis*.** Interactions occurring specifically in primary cell wall tissues are also indicated. Direct protein–DNA interactions involving activation or repression are represented using solid edges, while known regulatory relationships in which the mechanism is unclear are represented with dashed edges. Repressors are denoted with red edges. Protein–protein interactions are represented as ◊; question marks represent unidentified upstream TFs; overlapping edges (MYB46, MYB83) represent redundancy. Target genes are arranged semi-hierarchically according to known functions. The complete list of supporting literature used to construct the network can be found in Data sheet 1

A simplified representation of the SCW regulatory network is shown in Figure 1, which depicts the putative positions of associated TFs and their direct or indirect targets. We have also reconstructed a SCW-regulating protein–DNA and protein–protein interaction network from the *Arabidopsis* literature using BioTapestry (Longabaugh et al., 2005), showing cell type contexts where known (Figure 2). Aside from the indicated exceptions, we represent only direct protein–DNA interactions, as elucidated using yeast one-hybrid, electrophoretic mobility shift assay, chromatin immunoprecipitation, or post-translationally induced protoplast transactivation (see section Methodologies for the Study of SCW Transcriptional Regulation). Such interactions are referred to as direct regulation in this review. Finally, we provide as a supplementary file (Additional file 1) a more detailed network capturing the vast majority of demonstrated direct and indirect protein–DNA interactions and all known protein–protein interactions. This resource can be interactively visualized and manipulated with the freeware program Cytoscape (Shannon et al., 2003), and is accompanied by a list of the literature supporting each of the 435 captured interactions (Data sheet 1). To the best of our knowledge, this is the most exhaustive network representation compiled to date. The Cytoscape representation has several uses. First, it assists the generation of hypotheses related to biological function of poorly characterized proteins based on their interactions with known proteins. Second, additional attributes such as expression data may be integrated into the network to better understand network function and behavior. This is further enhanced by the fact that the network layout can easily be converted into built-in or customized views, and new interactions added as they are reported in the literature. In future, researchers may be able to use the network to provide priori structural information for the building of probabilistic causal networks that integrate diverse types of data, as performed in yeast by Zhu et al. (2012). Third, the network serves as a reliable basis for template-based construction of SCW transcriptional networks in sequenced non-model organisms (Babu et al., 2009).

At least three main tiers of TFs can be identified in the network that ultimately regulate a suite of structural genes involved in cellulose, hemicellulose and lignin biosynthesis, signal transduction, the cytoskeleton, programmed cell death and proteins with unknown functions (Figures 1, 2). We designated TF tiers from the bottom upwards, relative to a reliable reference point, i.e., the structural genes. A similar convention has been adopted before (Jothi et al., 2009). First-tier TFs are only known to directly regulate structural genes, second-tier TFs directly regulate first-tier TFs in additional to structural genes, and so forth. We stress that this assignment is not rigid and that TFs may be re-assigned, where possible, to a different tier as additional data arises. Furthermore, extensive feedback may occur between tiers.

SCW transcriptional networks in different cell types that synthesize SCWs are initiated by distinct, functionally redundant pairs of NAC proteins, which have been broadly referred to as secondary wall NACs (SWNs) (Zhong et al., 2010c) (Figure 2; third tier). Specifically, SCW deposition in xylary and interfascicular fibers (Mitsuda et al., 2007; Zhong et al., 2007b, 2008a) as well as silique valve endocarps and valve margins (Mitsuda and Ohme-Takagi, 2008) is redundantly regulated by NAC SECONDARY WALL THICKENING PROMOTING FACTOR1 (NST1) and SECONDARY WALL ASSOCIATED NAC DOMAIN PROTEIN1 (SND1). SND1 has also been referred to as NST3 and ANAC012 (Ko et al., 2007; Mitsuda et al., 2007; Mitsuda and Ohme-Takagi, 2008); to avoid confusion, we refer to this protein as SND1. In meta- and protoxylem vessels, a SCW deposition is regulated by VASCULAR RELATED NAC DOMAIN6 (VND6) and VND7, respectively (Kubo et al., 2005; Yamaguchi et al., 2008, 2010a; Zhong et al., 2008a). NST1 and NST2 are SCW master regulators in the endothecium of anthers (Mitsuda et al., 2005). To date, comparatively little data are available for the regulatory functions of NST2. MYB26 activates *NST1* and *NST2* in the endothecium through an as yet unknown mechanism (Yang et al., 2007), suggesting the existence of a fourth tier (Figure 2).

While the SCW master regulators in fibers, vessels, siliques and anther endothecia differ from one another, current data suggest that they regulate a common core transcriptional network (Figure 2). VND6/VND7 and NST2 regulatory functions largely overlap with those of SND1/NST1, but a number of targets are unique to VND6, VND7, or SND1 (Figure 2). Notably, vessel differentiation is distinguished from fiber development by strong VND6/VND7-mediated activation of genes involved in programmed cell death (PCD); in contrast, PCD gene activation by SND1/NST1 is weak (Ohashi-Ito et al., 2010; Zhong et al., 2010c) (Figure 2). A second notable difference is the fact that VND6/VND7 participate in a positive feedback loop with ASYMMETRIC LEAVES2/LATERAL ORGAN BOUNDARIES DOMAIN TFs ASL19 and ASL20 (Soyano et al., 2008), and VND7 additionally interacts with the transcriptional repressor protein VNI2 (Yamaguchi et al., 2010b) (see section Network Dynamics) which has not been identified in other cell types. VND7 also interacts with VND1, VND2, and VND3 (Additional file 1) which do not have clearly defined functions, whereas VND6 interacts primarily with itself and probably binds as a homodimer *in vivo* (Yamaguchi et al., 2008).

Third-tier SWNs directly regulate common second-tier MYB domain TFs *MYB46*, *MYB83*, and *MYB103*, NAC domain TFs *XND1* and *SND3*, and ASYMMETRIC LEAVES2/LATERAL ORGAN BOUNDARIES DOMAIN TF *ASL11* (Zhong et al., 2010c; Yamaguchi et al., 2011) (Figure 2). MYB46 and MYB83, which are functionally redundant, appear to form a common regulatory hub in the second-tier that directly regulate first-tier TFs *MYB6*, *MYB43*, *MYB52*, *MYB54*, *MYB58*, and *MYB63*, the functionally redundant trio *MYB4/MYB7/MYB32* (see section Network Dynamics), a C3H-type zinc finger gene *C3H14* and homeobox TF *KNAT7* (Ko et al., 2009; McCarthy et al., 2009; Nakano et al., 2010; Zhong and Ye, 2012) (Figure 2). *KNAT7* is directly activated by all the SWNs (Zhong et al., 2008a). In turn, KNAT7 represses cellulose, hemicelluloses and lignin biosynthetic genes directly or indirectly (Li et al., 2012a) (Figure 1). KNAT7-mediated repression is dependent on protein–protein interactions with MYB75, a weak transcriptional activator which has no known targets or direct regulators (Bhargava et al., 2010, 2013) (Figure 2). MYB20, associated with the regulation of lignin biosynthetic genes, is likely a first-tier candidate since it is an indirect SND1 target but downregulated in the *myb103* mutant (Zhong et al., 2008a; Öhman et al., 2012) (Figure 1). A number of novel bZIP, homeodomain, BEL1-like and zinc finger TFs that have not been linked to SCW regulation were also listed as MYB46/MYB83 direct targets (Zhong and Ye, 2012) (Additional file 1, Data sheet 1). Dominant repression of MYB52 and MYB54 result in reduced fiber SCW deposition (Zhong et al., 2008a). Enhanced drought tolerance in MYB52 overexpression lines (Park et al., 2011) suggests a pleiotropic role for this gene in both fiber development and abiotic stress response.

The first-tier TFs regulate various SCW biosynthetic genes although some members of the second-tier (MYB46, MYB61, and MYB83) and third tier (SND1, VND6, and VND7) also directly activate structural genes. BP, ATHB18, a C2H2-type zinc finger protein At3g46080, MYB20, MYB69, MYB79, MYB85, and the functionally redundant pair MYB58/MYB63 are known only to directly or indirectly regulate lignin biosynthetic genes (Mele et al., 2003; Zhou et al., 2009; Mitsuda et al., 2010), whereas BES1 is the only TF currently shown to bind to *cellulose synthase* (*CesA*) genes in both primary and SCWs (Xie et al., 2011) (Figures 1, 2). BP is a *KNOX* gene family member that maintains shoot apical meristems (Sanchez et al., 2012) and strongly represses lignification in inflorescence stems (Mele et al., 2003). MYB85 appears to specifically regulate the lignin pathway (Zhong et al., 2008a) and appears to be regulated by MYB46/MYB83 (Figure 1, Additional file 1). All other TFs regulate structural genes involved in the biosynthesis of more than one SCW biopolymer. SND2 has an unclear position in the network: it is known to be indirectly activated by SND1 (Zhong et al., 2008a), it is downregulated in the *myb103* mutant (Öhman et al., 2012), and appears to regulate genes related to signaling, hemicellulose and lignin polymerization in addition to the secondary wall *CesA* genes (Hussey et al., 2011; Öhman et al., 2012) (Figure 1, Additional file 1). Therefore, we have tentatively placed it in tier 1.

### Master regulators

SND1, NST1, NST2, VND6, and VND7 are considered master regulators of SCW formation because of their sufficiency for ectopic SCW deposition in some non-sclerified cell types when ectopically overexpressed (Mitsuda et al., 2005, 2007; Zhong et al., 2006; Yamaguchi et al., 2010a). By this definition, MYB family proteins MYB46, MYB83, and their direct target *C3H14* (Kim et al., 2012a), are also master regulators, despite occurring directly underneath SND1/NST1 and VND6/VND7 in the network (Zhong et al., 2007a; Ko et al., 2009; McCarthy et al., 2009). MYB83 is considered redundant with MYB46 since compromised functioning of both genes is required to visibly affect the phenotype (McCarthy et al., 2009). Arguably, MYB58 is a master regulator of the lignin pathway because overexpression causes ectopic lignification (Zhong et al., 2008a). Conversely, non-master regulators of SCW formation are recognized by subtle cell-specific phenotypes when overexpressed: for example, *SND2*, *SND3*, and *MYB103* lie downstream of master regulator SND1 (Figure 1), and their constitutive expression yields differences in SCW thickness only currently identified in fibers (Zhong et al., 2008b; Hussey et al., 2011). The factors rendering these TFs insufficient for ectopic SCW deposition are unclear, but a likely explanation is that auxiliary co-regulators are required for transcriptional activation or repression which are only expressed in the cells where a phenotype is observed. Discovery of these tissue-specific factors or protein complexes will advance the elucidation of the SCW transcriptional network.

Notably, the phenotypic importance of these master regulators does not correlate with their hierarchical position in the network: for example, *MYB46* and *MYB83* are subordinate to NST1 and SND1, but the double mutant of the subordinate pair yields a more extreme phenotype than the *snd1 nst1* double mutant (Zhong et al., 2007b; McCarthy et al., 2009). The genome-wide identification of direct gene targets of SND1 (Ko et al., 2007; Zhong et al., 2010c), VND6/VND7 (Ohashi-Ito et al., 2010; Zhong et al., 2010c; Yamaguchi et al., 2011) and MYB46/MYB83 (Zhong and Ye, 2012) have revealed key regulatory features of these master regulators. First, they do not preferentially activate TFs located in the first subordinate tier, such that the signal is relayed to successive tiers and ultimately to the structural genes at the bottom of the network. Rather, they directly regulate structural genes in addition to subordinate TFs (Figure 2). This pattern is consistent with the tendency of top and middle-tier TFs to act co-operatively in target gene regulation (Gerstein et al., 2012). Second, functional redundancy between proteins as assessed through mutant and complementation studies need not imply that redundant homologs regulate the same gene targets: although this might be true of MYB46 and MYB83 (Zhong and Ye, 2012), SND1 and VND6 share only ~50% of their target genes (Ohashi-Ito et al., 2010). SND1 and VND6/VND7 are quantitatively different in that PCD-related genes are upregulated strongly by vessel-associated VND6/VND7 but weakly, if at all, by fiber-associated SND1/NST1 (Ohashi-Ito et al., 2010; Zhong et al., 2010c) (Figure 2).

Induction of SND1 in undifferentiated transgenic *Arabidopsis* suspension culture cells is sufficient for smooth SCW deposition reminiscent of fibers, whereas induction of VND6 is sufficient for that resembling metaxylem vessels (Ohashi-Ito et al., 2010). Similarly the complementation of SCW deposition of fibers in the *snd1 nst1* double mutant by VND7 driven by the *SND1* promoter resulted in vessel-like patterning of the fiber SCWs (Yamaguchi et al., 2011). This suggests that SND1/NST1 and VND6/VND7 are sufficient for fiber- and vessel-specific differentiation. However, ectopic overexpression of SND1 only induced ectopic SCW deposition in particular cell types, with SCW patterning including smooth, banded, reticulated or helical deposition depending on the cell type (Mitsuda et al., 2005; Zhong et al., 2006). Poplar VND and NST homologs preferentially induce ectopic SCW deposition in hypocotyls, rather than leaves or roots, when constitutively expressed in *Arabidopsis* (Ohtani et al., 2011). Additionally, whilst all SWNs can transactivate the promoter of the PCD-related gene *XCP1* in protoplasts, the gene is not expressed in fibers under the control of SND1/NST1 (Zhong et al., 2010c). Together, these data suggest that whilst fiber- and vessel-associated SWNs preferentially confer SCW deposition patterns characteristic of these cell types, the action of other regulator mechanisms between cell types may modify their gene targets.

### Evolutionary conservation

The evolutionary history of SWN-mediated SCW regulation is not yet resolved. Although the moss *Physcomitrella* and primitive tracheophyte *Selaginella* possess multiple NAC proteins ancestral to the SWNs found in angiosperms, these proteins lack the extended C-terminal motifs found in derived SWNs (Shen et al., 2009; Zhao et al., 2010b; Zhu et al., 2012). Whilst their functions are currently unknown, it is thought that these progenitor SWN proteins were adapted for the regulation of SCW deposition in advanced vascular plants (Zhong et al., 2010a), mainly through the acquisition of C-terminal activation motifs, such as the WQ-box which is essential for SND1 transcriptional activation (Ko et al., 2007). There is strong evidence that these C-terminal expansions preceded angiosperm radiation (Shen et al., 2009).

The basis for the evolutionary conservation of functional redundancy between SND1-NST1, NST1-NST2, and VND6-VND7 pairs in different cell types in *Arabidopsis* and possibly other angiosperms is also poorly understood. Although postulated to be a backup mechanism to ensure SCW deposition ensues (Schuetz et al., 2013), a wealth of theoretical models have been proposed to explain the persistence of functional redundancy in higher organisms (Nowak et al., 1997; Krakauer and Nowak, 1999; Zhang, 2003). Redundancy appears to be a general characteristic of transcriptional regulators, as suggested by their underrepresentation amongst genes with single-copy status identified across 20 Angiosperms (De Smet et al., 2013). Interestingly, *Medicago* is the only angiosperm known to possess only one SWN, MtNST1. The *Mtnst1* mutant exhibits loss of fiber SCW deposition, reduced anther dehiscence and even defective guard cell functioning, but no apparent effect on vessels (Zhao et al., 2010a). Thus, *Medicago* appears to have dispensed of the redundant homologs and may serve as a suitable candidate for the study of the evolutionary persistence of functional redundancy in other groups.

Numerous examples of functional conservation between *Arabidopsis* SCW-regulating TFs and their homologs in a variety of plants suggest that the SCW transcriptional network is largely conserved in angiosperms. Functional orthologs of *Arabidopsis* SWNs and MYB46 have been experimentally verified in the monocots *Brachypodium distachyon*, *Zea mays*, and *Oryza sativa*, suggesting the establishment of the basic structure of the SCW transcriptional network at least prior to monocot-dicot divergence (Zhong et al., 2011a; Valdivia et al., 2013). Strong evidence also corroborates an *Arabidopsis*–like transcriptional cascade in woody angiosperm species. Whilst homologs of several TF candidates in Figure 1 have been linked to xylem development in hybrid aspen (Bylesjö et al., 2009; Courtois-Moreau et al., 2009) and *Acacia* (Suzuki et al., 2011), studies in *Populus trichocarpa* principally have demonstrated functional conservation of many SCW-regulating TF orthologs. A number of functionally redundant co-orthologs of SND1 from *P. trichocarpa*, referred to as wood-associated NAC domain TFs (PtrWNDs), are capable of ectopic SCW formation in *Arabidopsis* and can complement the *snd1 nst1* double mutant (Zhong et al., 2010b). *Populus* orthologs of TFs regulated by SND1 in *Arabidopsis* (Zhong et al., 2008a) are likewise regulated by the *Populus* PtrWNDs (Zhong et al., 2011b), and a functional ortholog of KNAT7 has been described (Li et al., 2012a). *Populus* PtrMYB3 and PtrMYB20 demonstrated similar master regulatory functions to their *Arabidopsis* homologs MYB46/MYB83 (McCarthy et al., 2009, 2010) and are sufficient for ectopic lignification in *Arabidopsis* (Zhong et al., 2010b). *Eucalyptus gunni* also possesses an SND1 homolog, EgWND1, that displays functional conservation with *Populus* and *Arabidopsis* SWNs (Zhong et al., 2010a, 2011b). EgMYB2, a close homolog of MYB46/MYB83 from *E. gunnii*, binds to promoters of lignin biosynthetic genes *EgCCR* and *EgCAD2* (Goicoechea et al., 2005) and can complement the *myb46 myb83 Arabidopsis* mutant, suggesting functional orthology with MYB46/MYB83 (Zhong et al., 2010a).

The high degree of conservation in SCW-associated TF function between *Arabidopsis* and woody plants suggests that studies in the former are of direct relevance to SCW formation in other herbaceous and woody plants. In support of this, a genome-wide survey of *cis*-regulatory sequence combinations in promoters of *Arabidopsis* and *Populus* found that over 18,000 combinations are shared between these organisms and that most of these combinations are functional (Ding et al., 2011). However, it is not yet clear whether network topology is equally conserved, and it is possible that *cis*-element evolution, which is both necessary and sufficient for network rewiring (Carroll, 2008), has occurred between species. In rice, for example, there appears to be functional divergence between an AP2/ERF TF known as SHINE (OsSHN), which has a SCW-regulatory function, and its closest *Arabidopsis* and barley homologs which regulate wax and lipid biosynthesis (Aharoni et al., 2004; Broun et al., 2004; Kannangara et al., 2007; Taketa et al., 2008). OsSHN is tightly co-expressed with homologs of SCW-associated TFs and biosynthetic genes. Interestingly, *Arabidopsis AtSHN1* was shown to directly repress rice homologs of *MYB58*/*MYB63*, *NST1*/*NST2*/*SND1*, and *VND4*/*VND5*/*VND6* when overexpressed in rice (Ambavaram et al., 2011). Rice, but not *Arabidopsis* plants, overexpressing *AtSHN1* showed increased sclerenchyma SCW thickness, decreased lignin and increased cellulose content (Kannangara et al., 2007; Ambavaram et al., 2011). The likely explanation for this phenotype is that, whilst the homologs of master regulators and lignin-associated TFs such as MYB85 and MYB58/MYB63 are repressed by AtSHN1, other TFs (including homologs of MYB20/MYB43) are upregulated which may specifically regulate cellulose deposition (Ambavaram et al., 2011). Together, this data suggests that the differing SHN targets in rice (monocots) and *Arabidopsis* (dicots) have evolved through changes in *cis*-element composition in their promoters, rather than the SHN DNA-binding domain, since AtSHN1 can switch from wax to SCW pathway regulation depending on the genetic background.

### DNA-protein interactions

TFs promote or inhibit transcription of target genes by binding to *cis*-elements in their promoters. General canonical binding sites for MYB and NAC domain TFs have been identified, and a number of *cis*-regulatory elements recognized by TFs involved in SCW regulation specifically have been described (Table 1). The *secondary wall NAC binding element* (SNBE) was discovered in the promoters of SND1 direct targets, existing as several related variants in target gene promoters (Zhong et al., 2010c). It consists of 19 nucleotides and is semi-palindromic, as demonstrated by reverse complementation (Table 1). NST1, NST2, VND6, and VND7 all recognize the SNBE consensus sequence, but the differential ability of SWNs and their orthologs to activate naturally occurring variants of this element suggests that particular SWNs will preferentially activate SNBE elements of different promoters (Zhong et al., 2010c, 2011a). The SNBE sequence is essential for SWN-mediated promoter activation, and *cis*-element copy number is correlated with the strength of promoter transactivation (Zhong et al., 2010c). Recently, SWN homologs in the monocot *Brachypodium* were also shown to recognise the SNBE (Valdivia et al., 2013). Wang et al. (2011) have identified a significantly more specific SNBE-like element bound by SND1, TACNTTNNNNATGA, which does not appear to be semi-palindromic (Table 1). Both the SNBE and SNBE-like elements appear superficially similar to the general NAC recognition sequence (NACRS), but neither contains the previously reported “canonical” CACG motif (Tran et al., 2004) (Table 1). It has recently been revealed that NACs possess some degree of flexibility when binding as dimers, allowing for one monomer to bind to a strong canonical DNA element and the other monomer to a low-affinity element a variable number of bases away (Welner et al., 2012). This may explain why SNBE is not a perfect palindrome.

**Table 1:** ***Cis*-regulatory elements that have been linked to SCW biosynthesis or which serve as general binding motifs for TF families involved in SCW transcriptional regulation**.

**Element**	**Functional classification**	**Bound TF**	**References**
Minimal NAC recognition sequence (NACRS; Tran et al., 2004) TCNNNNNNNA**CACG**CATGT (core sequence in bold)	Abiotic stress response	ANAC19/55/72 ENAC1	Tran et al., 2004
			Sun et al., 2011
Secondary wall NAC binding element (SNBE) (T/A)NN(C/T)(T/C/G)TNNNNNNNA(A/ C)(G/)N(A/C/T)(A/T) =(T/A)NN(C)(T/ /G)TNNNNNNNA(A/G/C)(G/A)N( N)(A/T)^*^	Secondary cell wall biosynthesis	SND1, NST1, NST2, VND6, VND7 BdSWN5	Zhong et al., 2010c Valdivia et al., 2013
TACNTTNNNNATGA	Secondary cell wall biosynthesis	SND1	Wang et al., 2011
Tracheary element-regulating cis-element (TERE) (Pyo et al., 2007) CTTGAAAGCAA	Secondary cell wall biosynthesis	Possibly VND6/VND7	Ohashi-Ito et al., 2010
AC elements (Lois et al., 1989; Sablowski et al., 1994; Hatton et al., 1995)	Secondary cell wall biosynthesis/lignin biosynthesis	MYB58, MYB63, EgMYB2. PtMYB4, PttMYB021, PvMYB4	Patzlaff et al., 2003; Zhou et al., 2009; Rahantamalala et al., 2010; Winzell et al., 2010; Shen et al., 2011; Zhong and Ye, 2012
AC-I (SMRE8): ACCTACC			
AC-II (SMRE4): ACCAACC			
AC-III (SMRE7): ACCTAAC			
SMRE consensus ACC(A/T)A(A/C)(T/C)	Secondary cell wall biosynthesis/lignin biosynthesis	MYB46/MYB83	Zhong and Ye, 2012
M46RE (A/G)(G/T)T(T/A)GGT(A/G) = (T/C)ACC(A/T)A(A/C)(T/C)^*^	Secondary cell wall biosynthesis/lignin biosynthesis	MYB46	Kim et al., 2012a
Element R GTTAGGT =ACCTAAC^*^	Disease resistance	MYB46	Ramírez et al., 2011
MYB binding site IIG (MBSIIG) G(G/T)T(A/T)GGT(A/G) =(T/C)ACC(A/T)A(A/C)C^*^	General MYB binding?	MYB15, MYB84 EgMYB2	Romero et al., 1998; Goicoechea et al., 2005; Rahantamalala et al., 2010
BSb	Cambium-specific expression	Unknown	Rahantamalala et al., 2010
CTGGTT			

* Reverse complemented forms of the sequence. AC-related elements are underlined to highlight similarities between them.

TE-specific expression may be mediated by the 11 base pair *tracheary element-regulating cis-element* (TERE) (Pyo et al., 2007). The element was identified in the promoters of 60 *Arabidopsis* genes upregulated during *in vitro* TE transdifferentiation (Kubo et al., 2005; Pyo et al., 2007). These included SCW-associated *CesA4* and *CesA7* promoters which were not identified as direct SND1 targets by Zhong et al. (2010c). It was suggested from protoplast transactivation experiments that VND6 and VND7 recognize the TERE elements of several SCW-associated genes (Ohashi-Ito et al., 2010; Yamaguchi et al., 2011). However, Zhong et al. (2010c) showed using electrophoretic mobility shift competition assays (EMSA) that VND6, VND7, and SND1 do not bind directly to the TERE element, and that most genes regulated by VND6 and VND7 do not contain recognizable TEREs (Ohashi-Ito et al., 2010; Yamaguchi et al., 2011). From *XCP1* promoter deletion experiments, Yamaguchi et al. (2011) postulated that the TERE is essential for basal transcription of VND7 targets, whilst their data supported the involvement of an additional element in enhancing VND7-mediated transactivation.

AC-rich elements are associated with various lignin biosynthetic genes (Raes et al., 2003) and are thought to be generally bound by MYB proteins (Zhao and Dixon, 2011). SCW-regulating MYB proteins from various taxa have been shown to bind AC elements (Table 1). The AC-like *cis*-element recognized by second-tier master regulators MYB46/MYB83 was independently identified by Zhong and Ye (2012; SMRE) and Kim et al. (2012a; MYB46RE), and the reported sequences are essentially identical following reverse complementation (Table 1, italicized). However, three of eight functional variants of SMRE correspond to AC-I, AC-II, and AC-III (Table 1) (Zhong and Ye, 2012) and MBSIIG, apparently a general MYB binding site recognized by *Arabidopsis* MYB proteins that are relatively distantly related from each other (Romero et al., 1998), is identical to SMRE/MYB46RE (Table 1). In fact, diverse *Arabidopsis* R2R3-MYB proteins bind to similar, if not identical, sequences due to highly shared recognition specificities (Romero et al., 1998; Prouse and Campbell, 2012). Despite this, MYB46RE is highly enriched amongst MYB46-regulated gene promoters compared to the genome frequency (Kim et al., 2012a), suggesting that MYB46RE/SMRE/MBSIIG may be specifically associated with MYB TFs involved in lignin and/or cell wall regulation. Specificity may be conferred by the requirement of multiple instances of the motif at a promoter, as is the case for EgMYB2 binding to the *EgCAD* promoter, or additional elements such as the linked BSb element that confers cambium-specific expression (Table 1) (Rahantamalala et al., 2010). Spatial expression specificity is discussed further in section Spatial Specificity of Fiber and Vessel Development. Notably F5H, which is required for the biosynthesis of S monolignols in Angiosperms, does not contain AC elements in the promoter region (Raes et al., 2003). Zhao et al. (2010b) found that *Arabidopsis* SND1 could directly activate the *Medicago F5H* promoter. However, Öhman et al. (2012) were not able to demonstrate transactivation of the *Arabidopsis* F5H promoter by SND1.

SCW-related canonical *cis*-elements have been identified *in vitro* through EMSA and *in vivo* through transactivation of naked DNA in GUS reporter constructs. However, accurate characterization of *cis*-elements, which should preferably resemble a probability distribution, will require genome-wide knowledge of occupied sites *in planta*. Available binding sites in a given cell type are heavily influenced by chromatin structure and composition, and TF specificity may be dependent on post-translational modifications and protein–protein interactions. Using ChIP-seq and its high-resolution derivative, ChIP-exo (Rhee and Pugh, 2011) (see section Methodologies for the Study of SCW Transcriptional Regulation), it will be possible to obtain statistical support for these motifs and assess single nucleotide dependencies, as has been done for MADS box TFs (Kaufmann et al., 2009, 2010).

### Network dynamics

Transcriptional networks are ultimately composed of small recurrent circuits known as network motifs, which are discrete patterns of interactions that occur more frequently than expected from randomized networks (Milo et al., 2002; Walhout, 2006; MacNeil and Walhout, 2011). In contrast to sensory networks, transcriptional networks regulating developmental processes tend to act slowly and can irreversibly trigger a transient developmental instruction. Negative and positive feedback loops and long cascades of transcriptional regulation are a prominent feature of developmental networks (see Alon, 2007 for review). Here, we explore network motifs and possible functions of putative modules in the SCW transcription network. Since network modules have no consensus definition (Dong and Horvath, 2007), we define them in this section as a group of connected nodes that collectively determines a pattern of target gene regulation distinct from the regulatory effect of each individual node on the target gene(s). These modules should be understood as teams of transcriptional regulators that co-operate to achieve an appropriate transcriptional response of a target gene(s) following a perturbation in the expression of an individual regulator in the module from steady-state levels.

**Figure 3:**
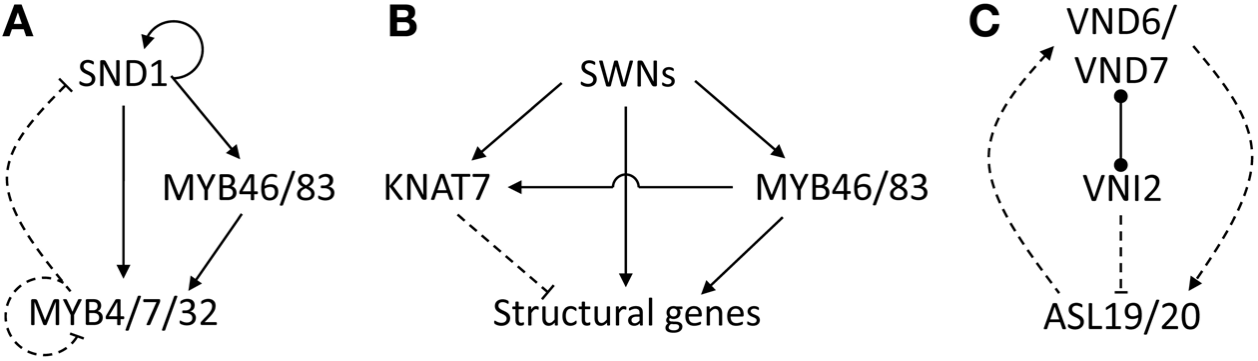
**Putative modules and motifs underlying SCW transcriptional regulation. (A)** Negative feedback loop regulating SND1. **(B)** Negative regulation of structural genes by KNAT7. **(C)** Positive feedback loop regulating VND6/VND7. Dashed edges indicate unknown molecular mechanisms of protein–DNA interactions. Arrows indicate positive regulation, blunt ends indicate negative interactions. Dumbbells represent protein–protein interactions. Refer to (see section Network Dynamics) for detailed discussion.

A negative feedback loop involving SND1, MYB46/MYB83 and a trio of repressors may prevent uncontrolled target gene activation during fiber SCW deposition. SND1 activates MYB32 directly, as well as indirectly through a coherent feed-forward loop involving SND1 targets MYB46/MYB83 which in turn activate MYB32 (Figure 3A). *MYB4* and *MYB7* are also targets of MYB46/MYB83 and have a conserved repression protein motif in common with MYB32 (Preston et al., 2004; Ko et al., 2009). Overexpression of a maize homolog of *MYB4* in *Arabidopsis* results in downregulation of the lignin pathway and a patchy SCW deposition phenotype in interfascicular fibers (Sonbol et al., 2009), supporting a repressive role for these proteins. SND1 and its poplar co-orthologs can self-activate their own promoters (Wang et al., 2011; Zhong et al., 2011b; Li et al., 2012b), an arrangement that is generally associated with a slow transcriptional response (Mejia-Guerra et al., 2012). MYB4, MYB7, and MYB32 in turn repress SND1 through an as yet unresolved mechanism (Figure 3A) (Wang et al., 2011). In addition, there is evidence from promoter transactivation experiments that MYB4, MYB7, and MYB32 repress their own promoters (Ko et al., 2009). Such negative autoregulation tends to accelerate transcriptional responses (Rosenfeld et al., 2002; Chalancon et al., 2012) and reduce transcriptional noise (Kærn et al., 2005; Alon, 2007). It could be postulated therefore that a combination of slow target gene activation by master regulator SND1, combined with a rapid MYB4/7/32-mediated negative feedback loop keeps SND1 activation in check, resulting in gradual target gene activation. This hypothesis is consistent with the prolonged lifespan and SCW deposition of fibers relative to vessels (Gorshkova et al., 2012).

In addition to negative feedback loops, a number of repressors of SCW deposition may help to prevent runaway structural gene activation or “fine-tune” their regulation. *XYLEM NAC DOMAIN 1* (*XND1*) is an SND1-activated NAC domain TF that may negatively regulate tracheary element growth (Zhao et al., 2008; Zhong et al., 2010c). No XND1 direct targets are currently known (Figure 2). Overexpression in *Arabidopsis* causes stunting, discontinuous or complete loss of xylem vessels, as well as a failure of xylem to undergo SCW deposition or PCD (Zhao et al., 2008). *KNAT7*, a class II *KNOX* gene that is also a direct target of SND1 (Zhong et al., 2008a), represses SCW deposition in xylary and interfascicular fibers through repression of cellulose, hemicellulose and lignin biosynthetic genes (Li et al., 2011, 2012a) (Figure 2). Surprisingly, *KNAT7* yields an *irx* phenotype in vessels of the null mutant (Brown et al., 2005; Li et al., 2012a), suggesting that KNAT7 may act as an activator in vessels (Schuetz et al., 2013). *SND1* and *KNAT7* form a type 1 incoherent feed-forward loop, such that SND1 activates structural genes as well as *KNAT7*, after which KNAT7 represses the structural genes once its protein has been synthesized (Figure 3B). This motif generates a pulse of target activation such that it reaches steady-state transcript levels faster than a simple regulation model, peaks and then declines to the stable target transcript abundance as the intermediate repressor becomes engaged (Alon, 2007). The response time of target gene activation is likely to be further accelerated by other TFs such as MYB46/MYB83 which also activate the structural genes. Thus, the putative module in Figure 3B is hypothesized to cause a rapid burst of structural gene transcript levels followed by a return to a steady state. KNAT7 additionally participates in protein–protein interactions with MYB75, a repressor of the lignin pathway that pleiotropically regulates anthocyanin biosynthesis (Bhargava et al., 2010, 2013), in addition to OFP1, OFP4, and MYB5 interaction (Wang et al., 2007a; Li et al., 2011; Bhargava et al., 2013). An interesting mechanism has been proposed whereby the differing fiber and vessel phenotypes observed in the *knat7* mutant depend on the composition and abundance of KNAT7-interacting proteins in different cell types (Li et al., 2012a). Bhargava et al. (2013) propose that KNAT7 forms a complex with OFP proteins and MYB75 to repress lignin biosynthetic genes in stems, whereas it forms a complex with TT8, MYB5, and MYB75 which represses SCW biosynthetic genes in the seed coat.

In contrast to the negative regulatory loop regulating SND1 in fibers (Figure 3A), VND6/VND7 master regulators of vessel SCW deposition are involved in a positive feedback loop with ASL/LBD family proteins (Iwakawa et al., 2002; Shuai et al., 2002). VND6/VND7 promote *ASL19/ASL20* upregulation through an unknown mechanism, and ASL19/ASL20 in turn promote VND6/VND7 upregulation such that they show similar expression patterns (Soyano et al., 2008) (Figure 3C). In addition, *ASL19* is downregulated by VNI2, a repressor that interacts with VND7 proteins to repress its function indirectly by competing with its heterodimerizing partners and possibly neutralizing VND7-mediated transcriptional activation (Yamaguchi et al., 2010b). Since VNI2 is sensitive to the ubiquitin proteosome pathway (Yamaguchi et al., 2010b), it has been postulated that the ASL19/ASL20/VND6/VND7 positive feedback loop promotes rapid and irreversible differentiation of vessel elements once VNI2 is proteolytically degraded (Ohashi-Ito and Fukuda, 2010).

A similar yet distinct mechanism to the VNI2-VND6/VND7 interaction has been documented in *Populus*. Recently, Li et al. (2012b) discovered a naturally occurring splice variant of a poplar SND1 co-ortholog *PtrSND1-A2*. The intron-retaining transcript variant, *PtrSND1-A2*^IR^, encodes a truncated protein lacking transactivation ability and a critical DNA-binding subdomain, but it retains its ability to form homo- and heterodimers. The dominant negative regulator represses *PtrSND1-A1*, *PtrSND1-B1*, and *PtrSND1-B2* by interfering with their self-activation abilities through the formation of non-functional heterodimers (Li et al., 2012b). The regulatory significance of this arrangement is not yet clear.

Network connectivity can only partially explain the behavior of a transcriptional network. Whilst regulatory hubs and modules may be identified from physical interaction networks, protein–DNA interactions alone may not accurately predict the outcome of target gene transcriptional regulation, which is complex and highly combinatorial (Spitz and Furlong, 2012). Kinetic data are required to mathematically model the dynamic behavior of a network (Bolouri and Davidson, 2002). New advances in network modeling allow for networks to be tested, quantified, and corrected (Sayyed-Ahmad et al., 2007). Time-course expression data in particular can capture dynamic properties of transcriptional networks that steady-state transcript measurements cannot (Nelson et al., 2004; Opper and Sanguinetti, 2010), and even time-course ChIP-seq data has been introduced into network models (Tang et al., 2012). *Arabidopsis* and *Zinnia* transdifferentiation systems are potentially useful models for generating time-course transcript data relating to SCW regulation, but existing time-course data (e.g., Kubo et al., 2005; Yamaguchi et al., 2011) lacks the temporal resolution to test and model the dynamic behavior of the SCW transcriptional network.

### Spatial specificity of fiber and vessel development

The preferential expression patterns of *SND1*/*NST1* and *VND6*/*VND7* in the *Arabidopsis* inflorescence and hypocotyl stems are remarkably fiber- and vessel-specific, respectively, and this expression pattern is consistent with the cell type showing a phenotype in loss-of-function mutants (Kubo et al., 2005; Mitsuda et al., 2007; Zhong et al., 2007b, 2008a; Yamaguchi et al., 2008). It is poorly understood how this cell-specific expression is achieved in xylem, but hypothetically cell type-specific signals direct *SND1*/*NST1* and *VND6*/*VND7* expression (Lucas et al., 2013). One tonoplast-localized, membrane-spanning transporter protein was found to influence *SND1*/*NST1* expression through an unknown mechanism in *Arabidopsis*: the *WALLS ARE THIN1* (*WAT1*) T-DNA mutant demonstrated a marked reduction in SCW formation in interfascicular and xylary fibers as well as a reduction in inflorescence stem growth, without otherwise affecting fiber cell specification (Ranocha et al., 2010). However, although *WAT1* transcripts are most prevalent in hypocotyls and inflorescence stems, the gene is almost ubiquitously expressed (Ranocha et al., 2010). In addition to the generally minor effect on overall growth in the *wat1* mutant, these characteristics of *WAT1* are in conflict with the idea that signals regulating the master regulators are themselves cell-type specific. In fact, the observation that widely expressed transcription factors may participate in cell type-specific regulatory roles (Neph et al., 2012) questions this expectation. The elucidation of a gene regulatory network of the *Arabidopsis* root stele showed that most TFs have a significantly broader expression pattern than their targets (Brady et al., 2011), suggesting that the SWN regulators may also be more broadly expressed than expected.

Examples of cell-to-cell signaling in the root may reveal clues to the specification of xylem cell types in vascular meristems. Protoxylem and metaxylem formation in the developing *Arabidopsis* root can be attributed to a gradient of class III HD-ZIP TFs such that high concentrations of these regulators promote metaxylem vessel formation and lower concentrations protoxylem vessel formation (reviewed in Caño-Delgado et al., 2010; Hirakawa et al., 2011). Specifically, the SHORT ROOT (SHR) TF is expressed in the developing stele, which moves into the endodermis to activate SCARECROW (SHR), both of which are involved in the endodermal expression of miRNA genes *MIR165A* and *MIR166B* (Carlsbecker et al., 2010). Diffusion of the resulting miRNAs from the endodermis toward the centre of the stele results in a decreasing concentration gradient (reviewed in Aichinger et al., 2012). Since miR165/166 post-transcriptionally inhibit HD-ZIP III TF *PHB*, an increasing gradient of *PHB* expression is created toward the centre of the stele, resulting in protoxylem formation at the stele periphery (i.e., low *PHB* concentration) and metaxylem vessel formation at the stele centre (i.e., high *PHB* expression) (Carlsbecker et al., 2010; Miyashima et al., 2011). Presumably, low *PHB* expression promotes *VND7* expression in protoxylem whilst high *PHB* expression drives *VND6* expression in metaxylem. However, this is yet to be investigated.

Whilst a miRNA concentration gradient model explains the formation of two distinct types of primary xylem cells in root, it cannot explain the pattern of fiber cells intercalated with vessel elements that is typically seen in secondary xylem. Such a system could be better explained, for example, by lateral polar auxin transport between adjacent cells, such that local foci of auxin maxima promote vessel differentiation whilst lower auxin concentrations promote fiber differentiation. Such a model is supported by the fact that, in root, lateral polar auxin transport determines the boundary between protoxylem and the procambium (reviewed in Milhinhos and Miguel, 2013), in stems the vessel density varies longitudinally as a function of the auxin concentration (reviewed in Sorce et al., 2013), and that the radial expression of auxin carrier genes in stems is non-uniform (Schrader et al., 2003). However, as discussed by Lucas et al. (2013), the localizations of auxin efflux proteins in stems and their distributions in fibers and vessels are currently unknown. It is most likely that a combination of hormones are involved: for example, the simultaneous presence of auxin, brassinosteroids and cytokinins was required for high expression of *VND6* and *VND7* (Kubo et al., 2005).

Some spatial specificity in SCW deposition can be explained by the presence of transcriptional repressors in non-sclerenchymatous cells. For example, *WRKY12* is expressed in stem pith and cortex, where it inhibits SCW formation by directly repressing SCW master regulators such as *NST2* (Wang et al., 2010) (Figure 2). The *wrky12* mutant shows ectopic SCW formation in the pith of both *Arabidopsis* and *Medicago* inflorescence stems, suggesting that repression, rather than activation, of SCW master regulators in specific cell types contributes significantly to their specific spatial expression. Interestingly, in *Populus* many *PtrWND* genes have surprisingly widespread expression, even in shoot apices and non-vascular parts of leaves (Han et al., 2011; Ohtani et al., 2011). It can be postulated that a transcriptional repressor or non-functional splice variant is expressed in non-vascular tissues and cells that binds to PtrWND proteins to prevent them from initiating ectopic SCW deposition, in a similar way to *PtrSND1-A2*^IR^ (see section Network Dynamics). Combined with the example above of the WRKY12 repressor that inhibits SCW initiation in some ground tissues in *Arabidopsis*, these data may point to an unexpected mechanism in which transcriptional activation of SCW deposition is a developmental program that is repressed in certain non-sclerified tissues, rather than simply induced in vascular tissues. Alternatively, co-factors required by these master regulators are not present in these non-vascular tissues, as evidenced by the failure of certain cells to ectopically deposit SCWs when the master regulators are overexpressed (see section Master Regulators).

The upstream regulators of SND1/NST1 and VND6/VND7 have not yet been reported, nor have the gene targets of xylem-regulating HD ZIPIII TFs (Figure 1), which are good candidates for SWN regulation. Knowledge of the SWN regulators will greatly enhance our understanding of how cell type-specific SCW transcriptional networks are initiated. The techniques used to infer TF function, and the interpretation of specific assays, are an important aspect of gene regulation studies. Moreover, recent advances in our understanding of eukaryotic gene regulation through projects such as the Encyclopedia of DNA Elements (encodeproject.org), necessitates an increasingly single cell-level understanding of transcriptional networks. We turn now to an evaluation of the molecular tools that have been used to study and infer SCW transcriptional networks, and which approaches will best support such studies in the future.

## Methodologies for the study of SCW transcriptional regulation

A number of techniques have been employed to study SCW-regulating TFs. We provide a summary of the advantages and challenges of common approaches used in the literature for TF functional annotation and SCW transcriptional network inference (Table 2). We have roughly arranged these techniques in increasing resolution of the regulatory information obtained in each; that is, increasing understanding of the *in vivo* direct gene targets of a given TF and its bound *cis*-element. Here, we discuss in greater detail the widespread use of reverse genetics and protoplast transfection approaches in model organisms in SCW regulation studies, and review approaches better suited to non-model organisms.

**Table 2:** **Summary of techniques used to study transcriptional regulatory networks**.

		**Advantages**	**Challenges**
*In vitro* (trans)differentiation systems (Fukuda and Komamine, 1980; Kubo et al., 2005; Oda et al., 2005)		Differentiation can be synchronized via hormonal induction A high proportion of cultured cells differentiate into TEs Time-course regulation of transcripts can be associated with developmental changes *Arabidopsis* suspensions can be stably transformed (Ohashi-Ito et al., 2010; Yamaguchi et al., 2010a) Provides temporal information to TE transcriptional regulation	Currently only developed in *Zinnia* and *Arabidopsis* Developmental *in planta* signals from neighboring cells are lost
Reconstruction from co-expression data		Co-regulated transcriptional modules can be identified Direct interactions can be inferred from data transmission theory (Basso et al., 2005) Provides functional sets of genes for *in silico cis*-element identification where genome is available	Transcriptomes from large numbers of diverse individuals, tissues and/or conditions required Guilt by association suffers from type 1 errors
Reverse genetics		Extensive catalog of mutant seedstocks available for *Arabidopsis* Phenotypic relevance of candidate TFs can be assessed Phenotypic effects of both gain- and loss-of-function mutants can be assessed	Lethal knock-out and repression lines cannot be analyzed Knock out lines not informative when TFs are functionally redundant Over-expression can lead to unexpected knock-on and dosage balance effects Generally suited to model organisms
Systems approaches	Systems biology	Molecular interactions can be quantified and contextualized Regulatory hubs can be identified and their regulatory effect assessed Novel candidates can be identified using multiple omics data which may be missed using one-dimensional data	Assumptions implicit to networks and modeling limit the biological accuracy of reconstructed networks Requires large numbers of good quality high-throughput data Generally more suited to model organisms
	Systems genetics	The effect of allele substitution on regulatory networks can be quantified Allows for the molecular basis of genetic associations to be understood Co-expression clusters and eQTL analysis may identify potential master regulators *Cis* and *trans* mechanisms of transcript regulation can be distinguished	Constrained by the degree of expression polymorphism within the population under study Large number of individuals required Condition-specific co-expression may escape detection Molecular basis of co-expression is unknown
Protein-binding microarrays (Mukherjee et al., 2004; Bulyk, 2007)		*Cis*-element sequences can be identified precisely Oligonucleotide arrays are applicable across all taxa *In vitro* results reportedly reflect *in vivo* binding	Purified GST-tagged protein may need to be functionally validated (e.g., EMSA) prior to assay Only dsDNA arrays can be used
Elecrophoretic mobility shift assay (EMSA)		Direct method to detect protein binding Can distinguish nucleotides essential for binding	*In vitro* method Low-throughput Heterologously expressed protein may not be soluble
Yeast 1-hybrid (Y1H) (Li and Herskowitz, 1993; Wang and Reed, 1993)		One of few gene-centered approaches available High-throughput robotic screening possible (Reece-Hoyes et al., 2011) Gateway-compatible short DNA fragments or long gene promoters can be used as baits (Deplancke et al., 2004) Custom stringency control possible	Prone to type 1 errors Yeast-expressed proteins may lack essential post-translational modifications Not suitable for TFs that require co-regulators to activate gene expression Cell-type context of interaction cannot be inferred
Transient protoplast transactivation systems		High-throughput (when combined with whole transcriptome analysis) Circumvents the need for stable transformation Little biological variation *In vivo* method Direct targets are inferred using post-translational induction in the presence of a protein synthesis inhibitor	Currently restricted to *Arabidopsis* mesophyll and *Populus* secondary xylem protoplasts (Wehner et al., 2011; Li et al., 2012b) Not suitable for TFs requiring tissue-specific co-factors (e.g., Bhargava et al., 2010) Possibility of false positives (misregulated genes) Cells are exposed to high levels of stress, which may influence the assay
Chromatin immunoprecipitation ChIP-on-chip (Ren et al., 2000) ChIP-seq (Barski et al., 2007) ChIP-exo (Rhee and Pugh, 2011) Nano-ChIP-seq (Adli and Bernstein, 2011)		High-throughput analysis of TF binding sites *In planta* method Can profile TFs that do not bind directly to DNA Canonical binding sites can be identified (esp. using ChIP-exo)	Critically dependent on antibody specificity and performance Limited ability to assay TFs exhibiting low or cell-specific expression Extensive optimization may be required for different tissues and organisms (Haring et al., 2007) Difficult to assign genes to TF binding sites, since not all binding sites are functional

Each technique is loosely arranged in order of increasing resolution of in planta protein–DNA associations.

Classical reverse genetics approaches employing overexpression and knock-out mutagenesis have been central to the functional annotation of SCW TFs in *Arabidopsis* and *Populus* (e.g., Zhong et al., 2007b; McCarthy et al., 2009; Grant et al., 2010). Direct or indirect targets of a TF subjected to knock-out or overexpression may be inferred under the premise that the transcriptional regulation of those targets is altered, leading to their differential expression relative to the wild type. However, we would like to highlight some problems associated with overexpression that have emerged in studies of SCW regulation, namely the level and site of overexpression.

SND1, now accepted as a master transcriptional activator of *Arabidopsis* SCW biosynthesis in fibers (Mitsuda et al., 2007; Zhong et al., 2007b, 2008a), was reported to suppress fiber SCW deposition when excessively overexpressed (Zhong et al., 2006). *SND2*, an indirect target of SND1, exhibited increased fiber SCW thickness when overexpressed and a mirrored reduction in SCW deposition in dominant repression lines (Zhong et al., 2008a). However, when our laboratory analyzed independent *Arabidopsis* lines overexpressing *SND2* (Hussey et al., 2011), we observed a decrease in fiber SCW deposition which we attributed to *SND2* transcript levels far-exceeding those reported in the previous study. Such phenomena could be explained by transcriptional squelching, defined as the repressive effect of a transcriptional activator beyond a certain threshold of abundance, due to the sequestration of interacting co-regulators or general transcription factors (Cahill et al., 1994; Orphanides et al., 2006). Alternatively a “dosage balance” mechanism (Birchler et al., 2005) holds that, for multi-subunit TF complexes, a relative increase in the abundance of one particular subunit does not lead to an increase in the yield of the assembled complex, but rather a stoichiometric reduction in the abundance of complete complexes and an increase in the abundance of non-functional sub-complexes (Birchler and Veitia, 2007, 2010). Together, these inconsistencies in functional studies of cell wall-related TFs suggest that overexpression differences may introduce indirect or even conflicting phenotypes.

Ectopic expression can also modify TF function. Regulating primary cell wall (PCW) deposition in the *Arabidopsis* root cap, three partially redundant TFs closely related to clade IIb NACs *NST1*, *SND1*, *VND6*, and *VND7* have been described, namely *SOMBRERO* (*SMB*), *BEARSKIN1* (*BRN1*), and *BRN2* (Bennett et al., 2010). When constitutively driven by the *35S CaMV* promoter, they are sufficient for ectopic deposition of lignified SCWs in several tissues, a phenotype resembling that of *NST1*, *VND6*, and *VND7* overexpression (Bennett et al., 2010). Since SCWs are not found in the root cap where the TFs normally function, ectopic expression resulted in a modification of the gene targets that *SMB* and *BRN1/2* naturally regulate, perhaps due to differences in co-regulators or other regulatory factors between tissues. This mechanism may also explain results reported by Bomal et al. (2008), where ectopic overexpression of the xylem-associated pine gene *PtMYB8* in spruce caused misregulation of flavonoid-associated transcripts, which have preferential expression in tissues that correspond to regions of low expression of native *PtMYB8* in pine.

The examples cited above of confounding effects due to the level and site of a candidate TF's expression provide compelling grounds to substantiate with additional evidence some of the conclusions arising from overexpression approaches. Such concerns have been echoed in a review of gain-of-function mutagenesis (Kondou et al., 2010). To avoid these problems, loss-of-function mutagenesis and non-transgenic approaches such as ChIP-seq may be more reliable. Although conventional mutagenesis is frequently unsuitable for SCW-regulating TFs due to the high degree of functional redundancy between homologs, the use of *c*himeric *re*pressor *s*ilencing *t*echnology (CRES-T; Hiratsu et al., 2003) has circumvented this problem, at least for transcriptional activators. In CRES-T, dominant loss-of-function transgenic plants overexpress a candidate TF fused to a hexapeptide dominant repression domain (Hiratsu et al., 2004; Mitsuda and Ohme-Takagi, 2008; Zhong et al., 2008a). The hexapeptide repressor opposes the transcriptional activation function at loci bound by the TF, in addition to the complementation that functional homologs may exert at those loci. Ectopic noise arising from overexpression may also be reduced by the use of tissue-specific promoters or laser capture-microdissection to capture only those cell types where the TF candidate is naturally expressed. Similarly, inducible expression may limit knock-on effects of long-term overexpression.

Promoter transactivation by an induced candidate TF in *Arabidopsis* mesophyll protoplasts (Wehner et al., 2011) has proved particularly useful in the identification of *Arabidopsis* gene targets, and may be used to complement approaches such as ChIP-seq which does not strictly indicate active target regulation (Table 2). The assay typically involves co-transfection with different plasmids, one harbouring a constitutively expressed candidate TF gene (the “effector”), a *promoter::GUS* reporter vector and a luciferase expression vector to allow for normalization of transfection efficiency. Inferring direct targets using this system is complicated by the potential ability of a candidate TF to induce transcription of an intermediate TF in the host cell that is responsible for activating a target gene. This has been addressed by translational fusion of the candidate TF to the regulatory region of the human estrogen receptor (Zuo et al., 2000). The chimeric protein is post-translationally induced by β-estradiol, allowing for an inhibitor of protein synthesis to be added to the system prior to induction to block the translation of intermediate TFs. The activated chimeric TF is then able to regulate transcription of target genes using the existing transcriptional machinery of the cell, and direct targets can be inferred with *promoter::GUS* RT-qPCR analysis (Zhong et al., 2008a; Zhou et al., 2009) or microarray analysis of the host cell transcriptome (Zhong et al., 2010c). For this reason, only those protoplast transactivation experiments that used post-translational induction were considered as evidence for protein–DNA interactions represented in Figure 2, to avoid the possibility that putative targets may have been indirectly regulated.

*Arabidopsis* mesophyll protoplasts have also been used to assess *in vivo* promoter transactivation by *Populus* and *Eucalyptus* TFs using *GUS* reporters fused to candidate promoters from these species (Zhong et al., 2011b). A genome-wide analysis of endogenous promoter transactivation of TFs from non-model species would require protoplasts derived from the same, or a related, species. Although the *promoter::GUS* approach suffers from much lower throughput, it mitigates the effects that the chromatin structure of the host protoplast may exert on the regulation of endogenous target genes. DNaseI hypersensitivity sites, an indicator of open chromatin marking most active TF binding sites, vary considerably across cell types (Thurman et al., 2012). Therefore, protoplasts should ideally be sourced from the same tissue in which a candidate TF is expressed. The recent report of isolation and transfection of *Populus* secondary xylem protoplasts (Li et al., 2012b) sets the stage for genome-wide analysis of poplar genes transactivated by poplar TFs involved in wood development.

Several approaches exist for *in vitro*, *in vivo*, and *in planta* analysis of TFs from non-model organisms that are not yet easily transformable (Table 2). Systems genetics allows for gene regulatory networks to be reconstructed by co-expression analysis across large numbers of segregating progeny (Ayroles et al., 2009). Microarray or RNA-seq expression data are obtained for tissues of interest from a structured segregating population. The addition of genetic markers allows for the identification of expression quantitative trait loci (eQTLs) (Jansen and Nap, 2001), which can be differentiated into those acting in *cis* or *trans*. Trans-eQTLs likely represent polymorphisms in transcriptional regulators, and due to their ability to affect expression of many genes, *trans*-eQTL “hotspots” may be mapped that contain significantly more eQTLs than the genome average. The combination of eQTLs and co-variation in transcript levels allows the prediction of causal relationships (Zhu et al., 2007) and candidate regulators (Drost et al., 2010) and is the basis on which regulatory networks can be constructed *a posteriori*, or hypothetical *a priori* networks tested (Kliebenstein, 2009). One considerable limitation of current systems genetics studies is the difficulty of studying the segregation of transcript abundance in specific cell types of organs. However, recent advances in obtaining high-throughput cell type-specific transcriptome data may make this challenge more feasible (Chitwood and Sinha, 2013).

Yeast one-hybrid (Y1H) has been widely used to identify TFs that interact directly with SCW-related promoters (Lin et al., 2010; Kim et al., 2012b). These assays can be performed either through direct cloning of candidate TF coding sequences and systematically testing interactions with different potential target promoters (Mitsuda et al., 2010), or via screening of cDNA expression libraries which have the advantage of discovering novel interacting proteins (Lopato et al., 2006). Recent advancements in Y1H screening, including smart pooling and robotics, have increased the generally low throughput of this technique (reviewed in Reece-Hoyes and Walhout, 2012). However, Y1H interactions occasionally fail independent validation assays. For example, although SPL8 was isolated from 20 of 72 yeast colonies showing a positive interaction with the *CCoAOMT1* promoter, it failed to activate the promoter in particle-bombarded *Arabidopsis* leaves (Mitsuda et al., 2010). A significant disadvantage of Y1H is that protein–DNA interactions which require cofactors or bind as complexes will not be identified (Table 2).

A poorly researched area in SCW transcriptional regulation is the symplastic movement of regulatory proteins between cells. It is well known that some TFs may move from cell to cell through plasmodesmata (Burch-Smith et al., 2011; Wu and Gallagher, 2012). Aside from the SHR example in section Spatial Specificity of Fiber and Vessel Development (reviewed by Kurata et al., 2005), in plants this has been found predominantly in meristems and involves mainly the KNOX (e.g., KNOTTED1) and MADS-box TF families (Zambryski and Crawford, 2000). However, at least one MYB-like protein is known to be non-cell-autonomous (Wada et al., 2002), and it is possible that some SCW-associated TFs act non-cell autonomously. This necessarily implies that the use of *in situ* hybridization, which has been widely used to study SCW-associated TF transcript abundance (e.g., Zhong et al., 2010b), may not accurately reflect a candidate TF's biological function. For species that can be stably transformed, TF movement can be tracked by fluorescent protein fusion experiments. For example, Kim et al. (2003) expressed GFP~KN1 fusion proteins (where ~ denotes a linker sequence) in mesophyll or epidermal cells using tissue-specific promoters, and compared the movement of GFP~KN1 between the mesophyll and the epidermis with that of free GFP and GFP fused to a viral movement protein. Microinjection of fluorescently labeled recombinant TFs into the cytoplasm of cells of interest can also be performed, but this approach is technically cumbersome and limited to larger cells (Lucas et al., 1995; Wang et al., 2007b). For non-model organisms, immunolocalization methods using an antibody against a TF of interest can be used to detect its presence *in planta*. Whilst low-abundance TFs may be difficult to detect using immunohistochemical methods, both alkaline phosphatase staining and immunogold labeling have been used to detect TF proteins at cellular and subcellular levels (Rodriguez-Uribe and O'Connell, 2006).

Chromatin immunoprecipitation combined with high-throughput sequencing (ChIP-seq) offers many advantages that are particularly suited to non-model organisms where genomic information is available (Table 2). It has been shown that even fragmented genome assemblies are acceptable for ChIP-seq read mapping (Buisine and Sachs, 2009), evading the need for genome assemblies on par with model plants. However, ChIP-seq in plants currently suffers from a lack of protocols for isolation of sufficient amounts of chromatin from individual tissues, and each tissue and species may require customized modifications to chromatin fixation, nuclei isolation and chromatin shearing (Haring et al., 2007). To our knowledge no ChIP procedures have yet been applied to developing xylem from woody stems, but a report of successful mapping of the ARBORKNOX1 TF in poplar vascular cambium using ChIP-seq (Andrew Groover, personal communication) sets the stage for its implementation in xylem. A range of improvements have been made to the basic ChIP-seq principle (reviewed in Furey, 2012), amongst them the ability to amplify sufficient amounts of ChIP DNA for Illumina sequencing from limited cell numbers (Adli and Bernstein, 2011). The latter is a particularly exciting advancement as it may allow for ChIP to be applied for the first time to plant tissues where chromatin yield is poor or where a TF's expression is low.

While these and other technologies advance—especially those involving second-generation sequencing—systems approaches to study SCW transcriptional regulation are still lacking. Systems biology attempts to integrate various high-throughput datasets into a holistic biological model, or achieve meaningful dynamic modeling of extensive biological data. Despite considerable progress in plant systems biology (Yuan et al., 2008) and the existence of several genome- and transcriptome-wide datasets relating to *Arabidopsis* SCW biosynthesis, few attempts have yet been made to integrate such data with other -omics platforms. The integration of metabolomic data into transcriptional networks, for example, can link modifications of TFs and their interactions to phenotypic outputs. Two model *Arabidopsis* studies, one using multiple knockout mutants of lignin biosynthetic pathway enzymes (Vanholme et al., 2012) and another analyzing five TF overexpression lines involved in glucosinolate biosynthesis (Malitsky et al., 2008), have integrated transcriptomic and metabolomic data to reveal novel aspects of metabolic pathway flux and regulation. In future, however, such analyses will have to be extended to cell-specific gene expression and interactions, especially in the field of transcriptional regulation. Overlaying cell type-specific expression profiles with Y1H and Y2H interaction data has been successfully achieved in the *Arabidopsis* root using enzymatic cell wall maceration and fluorescence-activated cell sorting of target cell protoplasts expressing a GFP marker (Brady et al., 2011). Another approach developed in *Arabidopsis* involves the purification of tagged nuclei from specific cells for transcriptome and ChIP-seq analysis (Deal and Henikoff, 2010). These and other innovations will undoubtedly contribute to a systems-level understanding of SCW regulation in the near future.

## Conclusions

In this review we aimed to provide a comprehensive summary of what is currently known about *Arabidopsis* SCW transcriptional regulation, highlighting current gaps in our understanding of the transcriptional network. We have also emphasized that an understanding of protein–protein interactions, spatial specificity and network dynamics (modules and hubs, regulatory motifs, and temporal regulation) is severely underdeveloped compared to what is known about the network's connectivity. The immediate goal of future research is to comprehensively identify the physical interactions (protein–DNA and protein–protein) involved in SCW transcriptional regulation. This includes the identification of not only interacting partners of known TFs, but also their cell-type context that might influence the functions of TFs in different ways. This goal will allow us to identify TFs and transcriptional modules that regulate genes involved in the biosynthesis of specific SCW biopolymers. This, together with systems approaches, will also reveal to what degree regulation of different genes and metabolic pathways is independent. Currently, only the lignin pathway seems to be specifically targeted by TFs such as MYB58, MYB63, and MYB85, and it may not be possible to uncouple the transcriptional regulation of cellulose and hemicellulose biopolymers. However, two recent studies have used components of the SCW transcriptional network to engineer plants with favorable biofuel properties by restoring vessel wall integrity in xylan (Petersen et al., 2012) and lignin mutants (Yang et al., 2013) or reinforcing polysaccharide deposition in fiber SCWs (Yang et al., 2013).

The ability to predict the regulatory outcome of perturbations in transcriptional networks through network modeling is invaluable to the field of biotechnology. A detailed knowledge of the strength of interaction for each edge connecting two nodes and a mathematical understanding of how the network responds to perturbations in expression, as well as genetic and environmental modulation, has not yet been attained. Systems biology experiments in *Arabidopsis*, for which knock-out lines are readily available to quantify network dynamics in response to genetic perturbations, will contribute extensively in this regard. For non-model organisms, Y1H and ChIP-seq are expected to be two key techniques used to identify protein–DNA interactions in the near future. However, systems genetics, which facilitates network reconstruction, modeling and quantification from perturbations caused by natural genetic variation, is gaining momentum in agronomically important species (Ingvarsson and Street, 2010; Mizrachi et al., 2012). Identification of trait QTLs and eQTLs additionally allow for the assessment of phenotypic impact of expression variation in TFs, the strength of association of TFs with regulons of co-expressed genes, and the ability to apply molecular breeding strategies to populations.

An understanding of the integration of intercellular signals, miRNAs, chromatin changes and temporal dynamics in transcription during xylem development remains a future challenge, marred by a limited understanding of regulatory mechanisms. For example, the occurrence of alternative splicing as a form of SND1 regulation in *Populus* (Li et al., 2012b) underscores an overlooked regulatory mechanism in SCW deposition, and there exists the possibility that certain RNA-binding proteins may participate in alternative splicing during xylogenesis. There is currently no data on cell type-specific chromatin modifications, DNA methylation or chromatin states during various aspects of fiber and vessel development that may influence availability of TF binding sites. We have no knowledge of the degree to which the SCW-associated TFs downstream of the HD-ZIP III TFs (Figure 1) are post-transcriptionally regulated by miRNAs, or of the transcriptional changes associated with the transitions between S1, S2, and S3 layer deposition in SCWs. Finally, the findings that fibers in close proximity to vessels show a vessel-like lignin composition (Gorzsás et al., 2011) and that lignification of tracheary elements may occur post-mortem due to monolignol transport from live cells (Pesquet et al., 2013) highlights the need to better understand the role of cell non-autonomous regulation of xylogenesis. Clearly there are plenty of opportunities for further study in this exciting field.

### Conflict of interest statement

The authors declare that the research was conducted in the absence of any commercial or financial relationships that could be construed as a potential conflict of interest.

## Supplementary material

Data sheet 1 **Excel spreadsheet of supporting literature for each protein-DNA and protein-protein interaction depicted in Additional file 1**.

Additional file 1 **Cytoscape file (.cys) of direct and indirect protein–DNA interactions and protein–protein interactions involved in *Arabidopsis* SCW transcriptional regulation**. Nodes indicate genes, edges indicate interactions. Red edges, known direct protein–DNA interactions; dark blue solid edges, known regulatory relationships of unknown nature; dark blue dashed edges; known indirect regulatory relationships; light blue edges, protein–protein interactions. Structural genes are indicated as square nodes, transcriptional activators as circular nodes, transcriptional repressors as diamond-shaped nodes, and transporter proteins (WAT1) as triangles. Nodes are color-coded according to known biological processes: blue, signaling; green, cellulose biosynthesis; yellow, hemicelluloses biosynthesis; black, programmed cell death; purple, lignin biosynthesis; white, unknown function. Transcriptional regulators colored in orange are involved in the regulation of more than one type of SCW biopolymer.
